# A Noted Worker at Oldham

**Published:** 1914-08-29

**Authors:** 


### A Noted Worker at Oldham.

THE LATE HENRY LISTER HARGRAVES.

The Oldham Royal Infirmary has suffered an irreparable loss by the death, on the 14th inst., of one of its two vice-presidents, in the person of Henry Lister Hargraves, J.P., at the ripe old age of eighty-seven. He passed peacefully away in his sleep at noon on the day mentioned, after a short illness.

Mr. Hargraves was elected a governor of the infirmary in the year 1885, and was elected (despite his protest at the time) as vice-president on July 25, 1912. For nearly thirty years he was one of the institution's most devoted workers and had a record attendance of 99 per cent. at its fortnightly meeting of the committee and weekly admission committees, besides a regular, almost daily, attendance spent in visiting the various wards of the infirmary, cheering and comforting the patients by kindly words and financial help to the needy. His death will be keenly felt by them.

Since his appointment in 1885 Mr. Hargraves superintended the purchase of all drugs and dressings used in the infirmary, a duty for which, as a retired chemist and druggist, he was pre-eminently qualified. He often expressed the pleasure he felt in saying that he and his father had dealt continuously with one firm, now known as the British Drug Houses, Limited, London, for over a century. He also claimed to be the oldest living member of the British Pharmaceutical Society, having been enrolled in the year 1841. Modest and unassuming to a degree, he went quietly along the pathway of life, doing without ostentation innumerable acts of charity and kindness, not only to the patients in the infirmary, but to hundreds of others in the borough of Oldham.

To the Oldham Royal Infirmary he gave from time to time fully £7,000 in sums ranging from £50 to £5,000 at a time. Other gifts included a grand piano for each of the nine wards; the investment of a sum to provide gold and silver medals to encourage the nurses of the institution, these being given to those who show the greatest aptitude in their profession in their respective years of training; the purchase of a new ambulance of the latest type, the cost of an entire renovation of the mortuary, and other benefactions too numerous to relate. Mr. Hargraves' generosity was not confined to the Oldham Royal Infirmary, for the Borough Art Gallery contains many valuable pictures given by him, together with playgrounds and open spaces for the young people, whilst the old folks' tea-parties were the delight of the aged of the town and district. As a member of the Society of "Friends," and in consonance with his daily life, Mr. Hargraves was interred quietly in Chadderton Cemetery, Oldham, on the 18th inst.

